# Heart-Kidney Transplants Linked to Better Outcomes Compared to Heart-Only Transplants in Children with Non-Dialysis-Dependent Advanced Chronic Kidney Disease

**DOI:** 10.21203/rs.3.rs-6674862/v1

**Published:** 2025-05-20

**Authors:** Ruchi Gupta Mahajan, Michael D Evans, Sarah J Kizilbash

## Abstract

Background Chronic kidney disease (CKD) significantly contributes to morbidity and mortality in children awaiting a heart transplant. The optimal approach to managing advanced CKD (stage 3b or higher) in children with heart failure—whether simultaneous heart-kidney transplant or heart-only transplant—remains unclear. Method Using the Scientific Registry of Transplant Recipients (SRTR), we identified all pediatric heart transplant recipients (age ≤ 18 years) with an estimated glomerular filtration rate (eGFR) of < 45 mL/min/1.73 m² at the time of transplant. We then divided the cohort into simultaneous heart-kidney recipients (n = 21) and heart-only recipients (n = 839). To evaluate patient and graft survival, we created a weighted comparison group of heart-only recipients using covariate-balancing propensity scores. Characteristics that were balanced included year of transplant, age at transplant, sex, race, HLA mismatch, cause of heart disease, previous heart transplant, and immunosuppression. We compared patient and heart graft survival between simultaneous heart-kidney and weighted heart-only groups using weighted Cox regression. Results We observed significantly higher patient survival (HR: 0.29; 95% CI: 0.1–0.81; p = 0.02) and heart graft survival (HR: 0.24; 95% CI: 0.08–0.67; p = 0.002) in simultaneous heart-kidney recipients compared to propensity-score-weighted heart-only recipients. Conclusions Compared to heart-only transplants, simultaneous heart-kidney transplants are associated with higher patient and heart graft survival in children with non-dialysis-dependent CKD stage 3b or higher.

### Introduction

The co-existence of heart and kidney dysfunction, termed cardiorenal syndrome, is being increasingly recognized in children. [1] It is mediated by organ crosstalk, whereby dysfunction in one organ induces dysfunction in the other, contributing to significant morbidity and mortality.[2–4] Heart transplantation is the definitive treatment for heart failure (5, 6), and preemptive kidney transplantation is the preferred treatment for children with kidney failure.[5] However, the optimal approach to managing non-dialysis-dependent chronic kidney disease (CKD) in children awaiting a heart transplant remains uncertain. The CKD caused by cardiorenal syndrome may improve with improved perfusion after the heart transplant, rendering a simultaneous kidney transplant unnecessary, or it may persist, resulting in significant morbidity and mortality in heart-only recipients.[6] A consensus statement endorsed by the American Society of Transplantation recommends considering simultaneous heart-kidney transplants for patients with a GFR below 30 ml/min/1.73 $m^2$. Additionally, the statement recommends considering select patients with an eGFR of 30–44 ml/min/1.73 $m^2$ for simultaneous heart-kidney transplants if they exhibit clear signs of progressive and/or irreversible chronic kidney disease (CKD), such as persistent proteinuria or small kidneys.[7] These recommendations are derived from adult data. While pediatric data demonstrate survival benefits of simultaneous heart-kidney transplants compared to heart-only transplants for patients on dialysis;[8] well-designed studies evaluating survival benefits for children with non-dialysis-dependent CKD are unavailable.

The optimal approach to managing non-dialysis-dependent CKD in children awaiting a heart transplant is complicated by the ethical dilemma posed by organ shortages. Since allocating multiple organs to one recipient limits the potential to save multiple lives from a single donor, well-designed pediatric studies are crucial to generating the necessary evidence to either support or challenge the practice of simultaneous heart-kidney transplants in children with non-dialysis-dependent CKD.

This study aimed to compare survival in children with non-dialysis-dependent advanced CKD (stage 3b or higher) who underwent a simultaneous heart-kidney transplant versus (vs.) those who received a heart-only transplant. Considering the significant post-heart transplant morbidity and mortality caused by CKD in children,[9] we hypothesized lower patient and heart graft survival with heart-only vs. simultaneous heart-kidney transplant among children with non-dialysis-dependent CKD stage 3b or higher (eGFR < 45 ml/min/1.73 $m^2$).

### Methods

The Institutional Review Board (IRB) of the University of Minnesota approved this study. The research complies with the International Society for Heart and Lung Transplantation (ISHLT) Statement on Transplant Ethics.

## Data source

This study used data from the Scientific Registry of Transplant Recipients (SRTR). The SRTR data system includes data on all donors, wait-listed candidates, and transplant recipients in the US, submitted by the members of the Organ Procurement and Transplantation Network (OPTN). The Health Resources and Services Administration (HRSA), U.S. Department of Health and Human Services provides oversight to the activities of the OPTN and SRTR contractors[10]. The data supporting this study's findings are publicly available from the SRTR.

### Study population

All heart transplant recipients aged 18 years or younger with an eGFR of less than 45 ml/min/1.73 m² at the time of heart transplant and who were transplanted between 1987 and December 31, 2022, were considered for inclusion in this study. We then divided the cohort into simultaneous heart-kidney recipients (n = 21) and heart-only recipients (n = 839). To evaluate patient and graft survival, we created a weighted comparison group of heart-only recipients using covariate-balancing propensity scores. Characteristics that were balanced included year of transplant, age at transplant, sex, race, HLA mismatch, cause of heart disease, previous heart transplant, and immunosuppression.

Children who were on dialysis at the time of heart transplant were excluded. Additionally, multi-organ recipients, other than heart-kidney, were excluded.

### Study variables

In this study, we examined the following variables: age at heart transplant, sex, race, pre-transplant dialysis, eGFR at the time of heart transplant (computed using the modified Schwartz equation as recommended by the International Society of Heart and Lung Transplantation [ISHLT] guidelines),[11] cause of heart failure, human leucocyte antigen (HLA) mismatch, prior heart transplant, induction and maintenance immunosuppression, cause of death, and cause of graft failure.

The type of heart transplant (simultaneous heart-kidney vs. heart-only) was the primary exposure of interest.

### Study Outcomes

The primary outcome of interest was patient survival. For patient survival, we followed the recipients from the date of heart transplant to the earliest of the date of death or the end of SRTR follow-up. We also evaluated heart allograft survival. For the latter, we followed the recipients from the date of heart transplant to the earliest of the dates of heart allograft loss, death, or the end of SRTR follow-up.

## Statistical Analysis

Balancing weights were estimated using the covariate balancing propensity score[12] to create a weighted comparison group of heart-only recipients with distributions of key prognostic characteristics similar to those of the simultaneous heart-kidney recipients. Characteristics that were balanced included year of transplant, age at transplant, sex, race, HLA mismatch, cause of heart disease, previous heart transplant, induction immunosuppression (polyclonal induction yes/no), and maintenance immunosuppression (Tacrolimus yes/no; mycophenolate yes/no; steroids yes/no). Covariate balance before and after weighting was assessed using absolute standardized mean differences (Fig. 1). The effect of balancing weights on statistical precision was summarized using the effective sample size. Participant characteristics were summarized using unweighted and weighted proportions and quantiles. Causes of death were compared using the weighted chi-square test. Patient survival and heart graft survival were compared between simultaneous heart-kidney and heart-only transplant recipients using weighted Cox regression. The proportional hazards assumption was tested, and there was no evidence of non-proportionality (PH test: patient survival, p = 0.54; graft survival, p = 0.64). Results of these models are reported using hazard ratios with 95% confidence intervals and p-values. Survival curves were constructed using the weighted Kaplan-Meier estimator. All analyses were performed in R version 4.4.2, including the WeightIt library version 1.4.0 for estimating balancing weights and the survey library version 4.4-2 for weighted estimators and tests.

### Results

Our final analysis cohort included 839 heart-only and 21 simultaneous heart-kidney recipients with non-dialysis-dependent CKD stage 3b or higher. However, the effective sample size after covariate-balancing propensity score weighting was 42 for the heart-only group.

### Recipient characteristics

Table 1 compares demographic and clinical characteristics between simultaneous heart-kidney and weighted heart-only recipients.

The median age at transplant for simultaneous heart-kidney recipients was 16 years (IQR: 13.0, 17.0). Sixty-two percent of simultaneous recipients were male, and 47.6% were white. The median eGFR of simultaneous heart-kidney recipients at the time of transplant was 29.6 (IQR: 20.6, 33.6) while the median eGFR of the weighted comparison group was 36.7 (IQR: 23.9, 40.8).

Fifty-two percent of simultaneous heart-kidney recipients received polyclonal antibody induction, 90.5% received tacrolimus, and 90.5% received mycophenolate for maintenance immunosuppression.

### Transplant outcomes

## Patient Survival

Figure 2 compares 10-year patient survival between simultaneous heart-kidney recipients and the weighted heart-only comparison group.

After weighting for year of transplant, age at transplant, sex, race, HLA mismatch, cause of heart disease, previous heart transplant, induction immunosuppression, and maintenance immunosuppression, we observed lower mortality hazards in simultaneous heart-kidney recipients compared to weighted heart-only recipients (HR: 0.29; 95% CI: 0.1, 0.81; p = 0.02) (Table 2)

However, we observed no difference in the causes of death between the two groups (p = 0.75) (Table 3).

## Heart Graft Survival

Figure 2 presents the graft survival curves for simultaneous heart-kidney and weighted heart-only recipients.

After weighting for year of transplant, age at transplant, sex, race, HLA mismatch, cause of heart disease, previous heart transplant, induction immunosuppression, and maintenance immunosuppression, we observed lower hazards of cardiac graft loss in simultaneous heart-kidney recipients compared to weighted heart-only recipients (HR: 0.24; 95% CI: 0.08, 0.67; p = 0.006) (Table 2).

## Discussion

This is the first population-based study to compare the outcomes associated with simultaneous heart-kidney vs. heart-only transplants in children living with heart failure and non-dialysis dependent CKD stage 3b or higher (eGFR of < 45 ml/min/1.73 $m^2$). We found significantly higher patient and heart graft survival rates in simultaneous heart-kidney transplant recipients compared to propensity-score-weighted heart-only recipients. Our study supports a GFR cut-off of 45 ml/min/1.73 $m^2$ for simultaneous heart-kidney transplant in children with non-dialysis-dependent CKD.

A retrospective SRTR study by Choudhry et al. of pediatric heart recipients involving 9245 heart-only and 63 simultaneous heart-kidney recipients found no benefit in survival with simultaneous heart-kidney transplants compared to heart-only transplants.[8] However, the comparison group in this study primarily consisted of patients with no or mild kidney dysfunction, as reflected by a median eGFR of 84.4 (IQR: 63.8, 110), limiting the applicability of these findings to patients with advanced CKD (eGFR < 45 mL/min/1.73 m²). When the authors stratified their analysis by dialysis status, they observed a survival benefit of simultaneous transplants in patients on dialysis only. Unlike our findings, they did not identify a benefit in those not on dialysis, likely due to the significantly better kidney function of the comparison group. Upon stratification by eGFR, they found a significant difference in survival between simultaneous and heart-only recipients with an eGFR of ≤ 35 mL/min/1.73 m² at the time of heart transplant. However, the ≤ 35 ml/min/1.73 $m^2$ group included patients on dialysis, making it likely that the dialysis status drove the association in this subgroup. Due to study design limitations, the data by Choudhry et al. are insufficient to rule out a survival benefit of simultaneous heart-kidney transplant in children with non-dialysis-dependent advanced CKD, highlighting the need for additional research. Our study addresses these limitations by excluding children on dialysis and restricting the analysis to those with an eGFR of less than 45 ml/min/1.73 m² in both the simultaneous and heart-only groups. With this approach, we observed improved survival in simultaneous heart-kidney recipients compared to heart-only recipients.

Our findings align with a recent study of 7,896 adult patients from the United Network for Organ Sharing database who had an eGFR of less than 60 mL/min and underwent simultaneous heart-kidney or heart-only transplants between 2005 and 2021. The study found that adults with an eGFR of less than 45 mL/min/1.73 m² had higher patient and heart graft survival rates with simultaneous heart-kidney transplants compared to heart-only transplants.[13] Another recent adult study, involving 1,124 simultaneous heart-kidney adult recipients and 12,415 heart-only adult recipients, found lower mortality in simultaneous recipients with an eGFR of less than 40 ml/min/1.73 m²; however, the study did not evaluate heart allograft survival.[14]

We observed significantly higher heart graft survival in simultaneous heart-kidney compared to heart-only recipients. The lower heart graft survival in heart-only recipients in the setting of advanced CKD is likely related to cardiorenal pathophysiology. Advanced chronic kidney disease contributes to post-heart transplant morbidity and mortality through hypertension, anemia, hyperphosphatemia, volume overload, and uremic toxins.[15–17] Furthermore, studies indicate fewer episodes of heart rejection and lower rates of coronary allograft vasculopathy in patients undergoing simultaneous heart-kidney transplants.[18] Reduced rejection rates are likely related to improved immune tolerance induced by the transplanted kidneys.[19,20] Since graft loss is closely tied to patient survival in the context of a heart transplant, higher graft survival in simultaneous heart-kidney recipients likely contributes to improved patient survival in this population compared to the heart-only group.

This is an important study as it provides evidence favoring simultaneous heart-kidney transplant in children with non-dialysis-dependent CKD characterized by eGFR of < 45 ml/min/1.73 $m^2$. Since heart graft failure is closely linked to mortality and heart retransplantation remains controversial due to decreased long-term survival and increased morbidity, [21] adopting an approach that maximizes post-transplant survival in children with heart failure is critical. The AST-endorsed consensus statement on simultaneous heart-kidney transplants proposes a safety net policy for heart-only recipients on chronic dialysis or with a persistent GFR ≤ 20 ml/min/1.73 m² for at least six weeks between days 30 and 365 post-transplant.[7] Under this policy, these recipients would receive priority for kidney transplantation from donors with a KDPI greater than 20%, aiming to ease the decision-making burden between simultaneous and heart-only transplants while preventing unnecessary dual organ transplants, given the scarcity of donor organs. However, these recommendations may not be beneficial for children and should be considered with caution. A retrospective United Network of Organ Sharing study of 10,030 pediatric heart transplants reported high waitlist mortality for pediatric heart recipients who were waitlisted for a kidney. Of the patients waitlisted, 18% died while waiting.[22] Furthermore, the study found that moderate-high sensitization at waitlisting (calculated panel reactive antibody ≥ 20%), possibly mediated by prior heart-only transplant, was associated with a lower likelihood of kidney transplant. Considering the challenges associated with subsequent kidney transplants in heart-only recipients and the clear survival benefit of simultaneous heart-kidney transplant in this population, we advocate for the latter in children with non-dialysis-dependent CKD stage 3b or higher. For patients with milder CKD, a heart-only transplant followed by a kidney transplant, if needed, is preferable given the organ shortage and the higher kidney allograft survival associated with sequential rather than simultaneous heart-kidney transplantation in this setting.[23]

This study has several limitations. Our sample size is small, with only 21 heart-kidney recipients. Due to the study's retrospective nature, we were unable to account for unmeasured confounders. We were unable to determine the proportion of heart-only recipients who improved their kidney function and the extent of the improvement following the heart transplant. Additionally, we used serum creatinine as a marker of kidney function, which may overestimate the GFR due to reduced muscle mass in the setting of a critical illness. Nonetheless, this study is important, as it is the first to demonstrate superior survival with simultaneous heart-kidney transplants in children with non-dialysis-dependent CKD stage 3b or higher, correlating with an eGFR of less than 45 ml/min/1.73 $m^2$.

In conclusion, simultaneous heart-kidney transplants are associated with higher patient and heart graft survival rates in children with non-dialysis-dependent advanced CKD (stage 3b or higher) compared to propensity-score-weighted heart-only transplants. Previous studies have demonstrated better outcomes of simultaneous transplants compared to heart-only transplants in children on dialysis. Our study extends these findings to children with non-dialysis-dependent advanced chronic kidney disease (CKD). Based on our study, we advocate that children with an eGFR of < 45 ml/min/1.73 $m^2$ should be considered for simultaneous heart-kidney transplants. Future studies should explore the clinical progression of CKD in pediatric heart-only transplant recipients, with a focus on identifying factors that may predict CKD improvement following transplantation.

## Abbreviations

Hazard ratios (HR)

Institutional Review Board (IRB)

Scientific Registry of Transplant Recipients (SRTR)

Organ Procurement and Transplantation Network (OPTN)

Health Resources and Services Administration (HRSA)

Kidney Donor Profile Index (KDPI)

Calculated Panel Reactive Antibody (cPRA)

Estimated Glomerular Filtration Rate (eGFR)

Human leukocyte antigen (HLA)

The International Society of Heart and Lung Transplantation (ISHLT)

Interquartile range (IQR)

Hennepin Healthcare Research Institute (HHRI)

International Society for Heart and Lung Transplantation (ISHLT)

## Declarations

Disclaimer

The data reported here have been supplied by the Hennepin Healthcare Research Institute (HHRI) as the contractor for the Scientific Registry of Transplant Recipients (SRTR). The interpretation and reporting of these data are the responsibility of the author(s) and in no way should be seen as an official policy of or interpretation by the SRTR or the U.S. Government.

**Author Contributions:** RM conceived the original idea for the study. RM and SK contributed to the design and implementation of the research. ME contributed to the data analysis. RM, ME, and SK participated in the writing of the manuscript.

**Funding Statement**: This research was supported by the National Institutes of Health's National Center for Advancing Translational Sciences grant UM1TR004405. The content is solely the responsibility of the authors and does not necessarily represent the official views of the National Institutes of Health's National Center for Advancing Translational Sciences.

**Conflict of Interest**: The authors declare that they have NO affiliations with or involvement in any organization or entity with any financial interest in the subject matter or materials discussed in this manuscript.

## Tables

Table 1: Study Cohort Characteristics

| Variables | Level | Heart-only transplant | Heart-only transplant, weighted | Simultaneous heart-kidney transplant |
|---|---|---|---|---|
| n | | 839 | 839 | 21 |
| Effective Sample Size | | 839 | 42.4 | 21 |
| Year of transplant (median [IQR]) | | 2004 [1998, 2010] | 2014 [2007, 2018] | 2012 [2007, 2019] |
| Age at transplant (median [IQR]) | | 0 [0, 10] | 16 [14, 17] | 16 [13, 17] |
| Sex n (%) | Female | 342 (40.8) | 16.2 (38.1) | 8 (38.1) |
| | Male | 497 (59.2) | 26.3 (61.9) | 13 (61.9) |
| Race n (%) | White | 655 (78.1) | 20.2 (47.6) | 10 (47.6) |
| | Asian | 17 (2.0) | 2.0 (4.8) | 1 (4.8) |
| | Black | 144 (17.2) | 18.2 (42.9) | 9 (42.9) |
| | Multiracial | 13 (1.5) | 0.0 (0.0) | 0 (0.0) |
| | Native | 6 (0.7) | 2.0 (4.8) | 1 (4.8) |
| | Pacific | 4 (0.5) | 0.0 (0.0) | 0 (0.0) |
| HLA mismatch (median [IQR]) | | 5 [4, 5] | 5 [4, 5] | 5 [4, 5] |
| eGFR at heart transplant (median [IQR]) | | 33.2 [24.8, 40.0] | 36.7 [23.9, 40.8] | 29.6 [20.6, 33.6] |
| Previous Heart Transplant n (%) | No | 746 (88.9) | 18.2 (42.9) | 9 (42.9) |
| | Yes | 93 (11.1) | 24.2 (57.1) | 12 (57.1) |
| Cause of heart failure n (%) | Congenital Heart Disease | 549 (65.4) | 16.1 (38.1) | 8 (38.1) |
| | Coronary Disease | 9 (1.1) | 1.2 (2.8) | 0 (0.0) |
| | Dilated Cardiomyopathy | 229 (27.3) | 20.1 (47.5) | 10 (47.6) |
| | Hypertrophic Cardiomyopathy | 18 (2.1) | 1.7 (4.0) | 1 (4.8) |
| | Other | 13 (1.5) | 2.3 (5.4) | 0 (0.0) |
| | Restrictive Myopathy | 19 (2.3) | 0.9 (2.0) | 2 (9.5) |

| | | | | |
|---|---|---|---|---|
| | Valvular Heart Disease | 2 (0.2) | 0.1 (0.3) | 0 (0.0) |
| Polyclonal antibody induction n (%) | No | 517 (61.6) | 20.2 (47.6) | 10 (47.6) |
| | Yes | 322 (38.4) | 22.2 (52.4) | 11 (52.4) |
| Tacrolimus n (%) | No | 490 (58.4) | 4.0 (9.5) | 2 (9.5) |
| | Yes | 349 (41.6) | 38.4 (90.5) | 19 (90.5) |
| Cyclosporin n (%) | No | 531 (63.3) | 38.7 (91.3) | 20 (95.2) |
| | Yes | 308 (36.7) | 3.7 (8.7) | 1 (4.8) |
| Mycophenolate n (%) | No | 407 (48.5) | 4.0 (9.5) | 2 (9.5) |
| | Yes | 432 (51.5) | 38.4 (90.5) | 19 (90.5) |
| Azathioprine n (%) | No | 507 (60.4) | 41.0 (96.8) | 20 (95.2) |
| | Yes | 332 (39.6) | 1.4 (3.2) | 1 (4.8) |
| mTOR inhibitor n (%) | No | 817 (97.4) | 41.8 (98.7) | 21 (100.0) |
| | Yes | 22 (2.6) | 0.6 (1.3) | 0 (0.0) |
| Maintenance steroids n (%) | No | 285 (34.0) | 4.0 (9.5) | 2 (9.5) |
| | Yes | 554 (66.0) | 38.4 (90.5) | 19 (90.5) |

Table 2: Causes of Death

| Cause of Death | Heart-only transplant | Heart-only transplant, weighted | Simultaneous heart-kidney transplant | p-value, weighted heart-only vs. simultaneous |
|---|---|---|---|---|
| Cardiovascular | 85 (20.2) | 14.2 (23.0) | 1 (25.0) | 0.751 |
| Cerebrovascular | 20 (4.8) | 3.8 (6.1) | 0 (0.0) | |
| Graft failure | 101 (24.0) | 11.8 (19.2) | 0 (0.0) | |
| Infection | 46 (10.9) | 4.8 (7.8) | 1 (25.0) | |
| Malignancies | 17 (4.0) | 10.1 (16.3) | 1 (25.0) | |
| Pulmonary | 27 (6.4) | 11.7 (18.9) | 0 (0.0) | |
| Other | 98 (23.3) | 3.3 (5.3) | 1 (25.0) | |
| Unknown | 27 (6.4) | 2.1 (3.4) | 0 (0.0) | |

## Figures

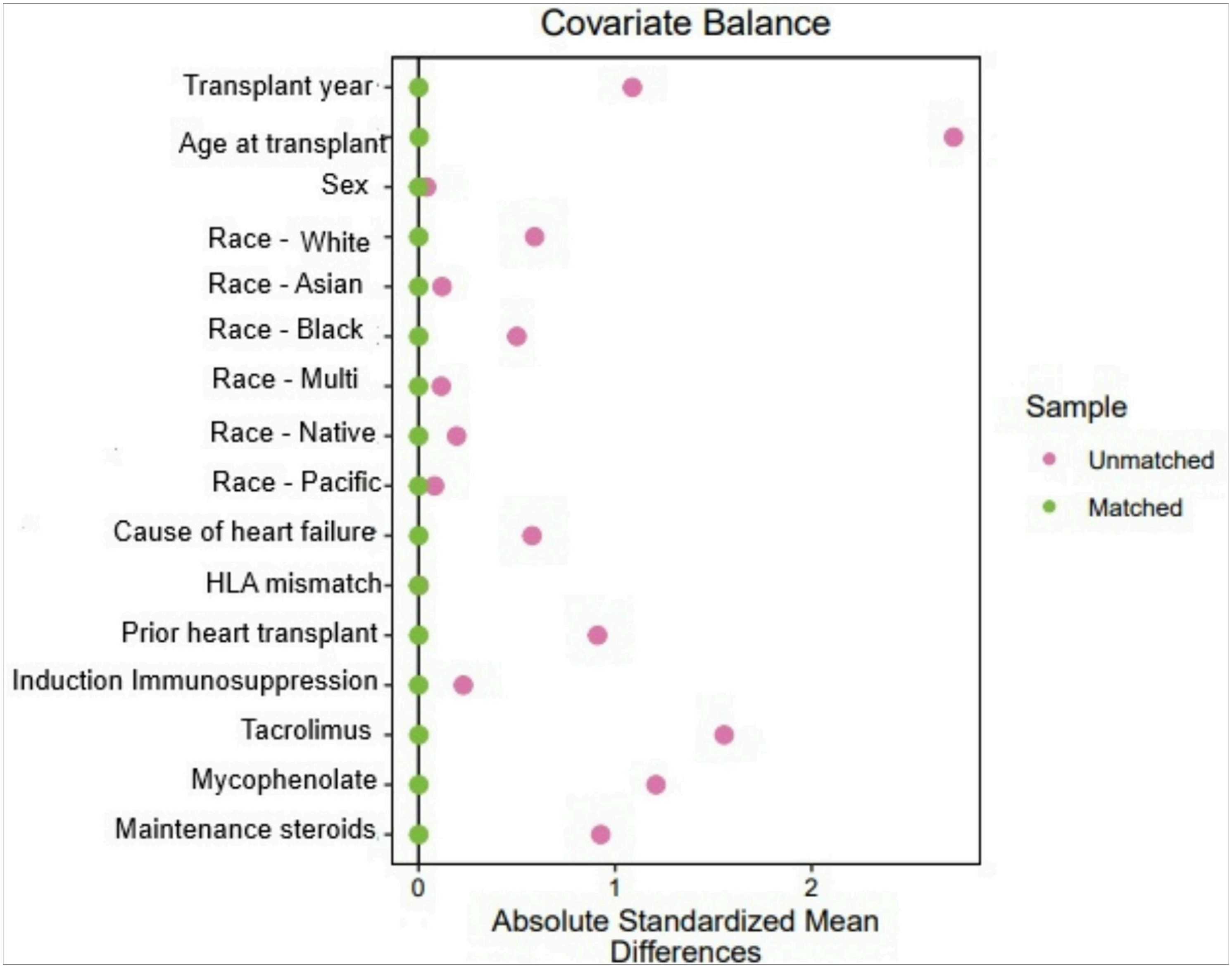

#### Figure 1

#### Covariate Balance

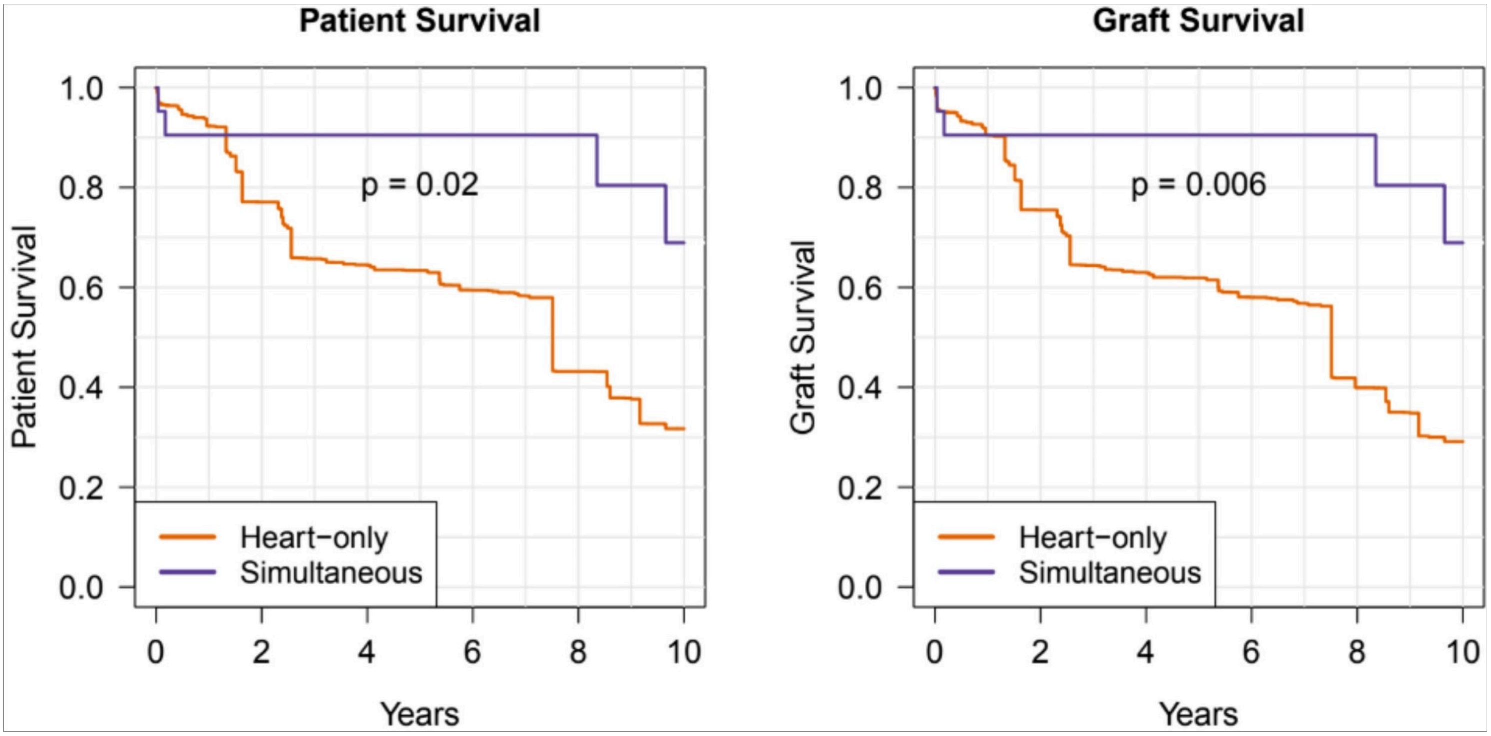

Figure 2

10-year Patient Survival and Heart Graft Survival in Simultaneous Heart-kidney and Weighted Heart-only Recipients
